# Editorial: Progress in Pediatric Urology in the Early 21st Century

**DOI:** 10.3389/fped.2019.00349

**Published:** 2019-08-20

**Authors:** Ricardo González, Barbara Magda Ludwikowski

**Affiliations:** Department of Pediatric Surgery and Urology, Kinder- und Jugendkrankenhaus AUF DER BULT, Hanover, Germany

**Keywords:** Pediatric Urology, Bladder Exstrophy, Neurogenic incontience, Laparoscopic surgery, Enterocystoplasty, tissue engineering, pediatric urolithiasis, bladder dysfunction

The intention of this Research Topic was to compile a collection of articles outlining areas of progress in pediatric urology since the onset of the twenty-first century. Some important topics, such as DSD, proximal hypospadias, and robotic assisted surgery were deliberately excluded since they have been the subject of recent Research Topics in Frontiers in Pediatrics. In the end we chose to cover laparoscopy, bladder exstrophy, urolithiasis, urethral strictures, and management of neurogenic incontinence, tissue engineering and the use of bowel in reconstruction. Finally, we were privileged to enlist Prof. Philip G. Ransley to express his thoughts on the future of the specialty. All of the senior authors of the articles included have an impressive track record on their fields. We are sure the readers will profit from reading this collection.

## Applications of Laparoscopic Surgery in Pediatric Urology

Szavay reviewed the topic of laparoscopic procedures in the pediatric urinary tract. Laparoscopic surgery, and reconstructive surgery in particular is a late twentieth and early twenty-first century development in pediatric urology. Beginning with the publication of the first pediatric laparoscopic pyeloplasty (LP) in 1995, the last 24 years have seen this procedure become the first choice of many surgeons to treat ureteropelvic obstruction in children of all ages. Mastery of laparoscopic suturing techniques requires good mentoring, a rather long learning curve and most of all the conviction that the effort is worth pursuing. The advantages of minilaparoscopy (using 3 mm working ports and a 5 mm umbilical port for the camera and to introduce the sutures) are not only cosmetic but results in superior outcomes with a negligible reoperation rate. Rapid recovery and minimal morbidity are the hallmarks of mini LP. Of course the availability of robot-assisted procedures with allegedly equal outcomes and much easier to master, has lured many surgeons away from learning the more demanding mini LP. Nevertheless, the need for 4 ports, the much larger trocars and the infinitely higher cost of robotic assisted surgery, not to speak the limited availability outside the more affluent centers, makes the learning of mini LP procedures a must for training the next generation of pediatric urologists.

The review by Szavay is limited to transperitoneal procedures. We do not consider this a shortcoming since in our practice, retroperitoneoscopic procedures have no place. Others continue to use retroperitoneoscopy arguing that in traditional urological open procedures the retroperitoneal approach is preferred. Theoretical advantages include less risk of injury to intraperitoneal structures and of development of adhesions. The price to pay is operating with the patient in the flank position, the use of larger trocars or incisions to create the working space,reduced visibility and a restricted working space in small children. In practice, after hundreds of cases of transperitoneal renal procedures, we never had a visceral or vascular injury and in reoperative cases, no or minimal adhesions are observed.

Other, both ablative and reconstructive procedures are well reviewed in this article.

## Recent Trends in the Management of Bladder Exstrophy: The Gordian Knot Has Not Yet Been Cut

This article from authors from Regensburg, Germany, led by Promm and Roesch focuses on the problem of the initial management of bladder exstrophy. With a thorough review of the literature, the authors tend to advocate a delayed closure, complete repair (including the epispadias) and no osteotomies. We wholeheartedly agree with their conclusions with minor exceptions. The advantages of delayed primary repair are obvious. The procedure need not be treated as an emergency, the best team of surgeons and anesthesiologists can participate and the failure rate in terms of dehiscence is not increased compared to neonatal closure. Also the waiting time gives the parents to seek other opinions and choose the center of their liking for the primary repair. We agree that osteotomies are not helpful in classical exstrophy cases since the initial success of a correctly done closure without osteotomies approaches 100% and there is no good evidence in the literature that either continence or sexual function are improved by them. Although we always do a complete repair in females, we have abandoned the complete repair in males in favor of closing the bladder and proximal urethra at the initial closure leaving the epispadias repair for a second operation. In our opinion, this method reduces the risk of penile ischemic damage. The authors of this review conclude that: “Further, clinical research should focus on multi-institutional collaborative trials to determine the optimal approach.”

## Effectiveness of Prenatal Intervention on the Outcome of Diseases That Have a Postnatal Urological Impact

Authors from Berlin and Hanover contributed a minireview on the published articles reporting outcomes of prenatal intervention for diseases that have an urological impact after birth (Bañuelos et al.). The review focuses mainly on LUTO, congenital adrenal hyperplasia, and myelomeningocele. The published reports provide evidence that in severe fetal bladder outlet obstruction in the second trimester, effective and lasting decompression allows survival in an otherwise almost invariably lethal situation, mainly thanks to improved lung development. It remains unclear if renal or bladder function are improved in the long term. Problems continue to be related to the near impossibility of conducting prospective randomized trials and shortcoming with instrumentation. Whether the time proven shunting with its many potential complications will eventually be replaced by fetoscopic procedures remains to be seen. Available information suggests that in the long term renal function is not improved (1) therefore the decision to intervene in the fetus with LUTO remains up to very well informed parents.

The fetal closure of myelomeningoceles seems to reduce the incidence of hydrocephalus and thus the need for shunting, an important cause for long term morbidity and mortality in spina bifida patients. Published reports offer conflicting results regarding the potential benefits of fetal closure with regards to bladder function. The answer will become clear in time when large numbers the operated patients reach mid childhood and bladder function can be evaluated more accurately than in infants.

Finally, the prenatal treatment with steroids of female fetuses at risk for being affected by congenital adrenal hyperplasia (with a positive family history) is entering a new era thanks to the possibility of making the diagnosis and determining the sex of the fetus on maternal blood sample before an amniocentesis can be performed. This will obviate the need to treat a large number of fetus who do not treatment either because they are not affected or males, since to be effective to prevent virilization, dexamethasone needs to be started before the 8th gestational week.

## Update on Surgical Management of Pediatric Urolithiasis

Sultan et al. from Karachi wrote an exhaustive review of current interventions to treat pediatric urolithiasis. They describe in detail current minimally invasive techniques used to treat stone in children. The strong point of this article, which reflects the enormous experience of the authors at the Sindh Institute for Urology and Transplantation, is the author's conclusion that: “Thus this manuscript guides how to select the least invasive option for an individual patient, considering age and gender; stone size, location and composition; facilities and expertise available.”

## Use of Bowel in Pediatric Reconstruction

The group from Manheim led by Stein et al. wrote an excellent review about the use of bowel in pediatric Urology. Despite the optimistic view that the use of bladder augmentation is becoming rare thanks to the success of non-operative methods to deal with diminished bladder capacity and compliance, centers dealing with large numbers of patients with spina bifida and bladder exstrophy still perform bladder augmentations or continent diversions rather frequently. As is the case with many reconstructive procedures, past experience and different interpretation of published data often leads to different practices by different surgeons. We personally use preferentially the sigmoid colon unless there are compelling reasons not to. The concept that the urodynamic results using sigmoid are not as good as with ileum stems from the popularization in some textbooks of incorrect methods to reconfigure the colon. When the sigmoid is reconfigured as Goodwin described the reconfiguration of the small bowel (2), the results are excellent with the additional advantage the use of sigmoid colon does not cause any potential metabolic or digestive problems and the incidence of post-operative bowel obstruction caused by adhesions is lower (3). We continue to avoid the use of the ileocecal segment also because its elimination from the gastrointestinal tract causes an acceleration of the intestinal transit that may impair fecal continence in patients with neurogenic bowel (4, 5).

The authors are correct in stating that the use of seromuscular colocystoplasty lined with urothelium has not gained wide acceptance. However, despite limited indications it plays an important place in the armamentarium of some pediatric urologists (6) and it avoids the potential metabolic consequences of intact bowel segments in properly selected cases.

## Traumatic Posterior Urethral Strictures in Children and Adolescents

Podesta and Podesta from Buenos Aires were asked to review the topic of pediatric traumatic posterior urethral strictures based on their vast experience and their published work. The review gives a comprehensive overview of the surgical options to correct traumatic posterior urethral strictures in children based on anatomical findings. These lesions, fortunately rare in the western world, are more common in other parts of the world. The authors recommend a classical approach to the initial management with suprapubic cystostomy and delayed repair for complete disruptions. They clearly indicate situations in which a primary realignment is recommended. They clearly describe the perineal approach, maneuvers to gain anterior urethral length and the indications for the transpubic approach. Although one of us has used and published experience with endoscopic reestablishment of urethral continuity and the classical transpubic approach described by Pierce and Waterhouse several decades ago, we now use primarily the perineal approach and now fully subscribe to the authors view that: “the progressive perineo-abdominal partial transpubic anastomotic repair has advantages over the isolated perineal anastomotic approach in patients with “complex” PFPUDD. This approach provides wider exposure and facilitates reconstruction of long or complicated posterior urethral distraction defects.”

## Anterior Urethral Strictures in Children: Disease Etiology and Comparative Effectiveness of Endoscopic Treatment vs. Open Surgical Reconstruction

The pediatric urology group in Hamburg (Vetterlein et al.) addressed the topic of anterior urethral strictures in children. In pediatrics, most anterior urethral strictures are iatrogenic or traumatic. The important message of this review is that urethroplasty is far superior to endoscopic procedures are rather ineffective in the long-term. They reach the important conclusion that open surgical reconstruction: “should be preferred to avoid multiple, repetitive interventions.” We wholeheartedly agree with this conclusion.

## Surgical Management of Neurogenic Sphincter Incompetence in Children

Authors from Hanover and Berlin (Ludwikowski et al.) contributed a literature review of results of surgical methods to treat neurogenic sphincteric incontinence. They conclude that injection of bulking substances to the bladder neck or proximal urethra as well as surgical methods to reconfigure the bladder outlet are of little practical value. Effective published methods include bladder neck sling in girls, artificial urinary sphincter implantation in both sexes and bladder neck closure. Unfortunately, as also reported by others, the level of published evidence is low.

## Tissue Engineering in Pediatric Bladder Reconstruction—The Road to Success

Horst et al. from Zurich describe the current state of research on tissue engineered bladders. This and many other groups are making steady but painfully slow progress toward reaching the goal of creating tissue suitable for use as a bladder wall substitute. The obstacles are formidable particularly relating to vascuarization and innervation of the implant. The transfer of tissue from the laboratory construct to a large animal continues to pose enormous challenges. Unfortunately the much publicized trial (7) which ultimately failed was directed at the wrong population. Obviously, the problem with myelomeningocele is the central and peripheral nervous system, not the target organ, the bladder. Even if a normal bladder could be constructed in the laboratory in this patient population it would have no greater chance of success than implanting a new leg that has abnormal innervation. Nevertheless, these research efforts are certainly worth pursuing since the knowledge acquired is enormous and will eventually yield useful clinical applications.

## Diagnosis and Management of Bladder Dysfunction in Neurologically Normal Children

The group from Salvador de Bahia led by Fuentes et al. presented a review of this topic in a clear and detailed fashion. Non-neurogenic bladder dysfunction is one of the most frequent problems confronting the pediatric urologist in everyday practice and is a confusing topic for the inexperienced. This article is very didactic and should serve as a guide for many starting a pediatric urological practice.

## The Future of Pediatric Urology

Ransley with decades of experience and a leader in the field, shares with us his view of our specialty in the decades to come with the inevitable progress in robotic surgery and artificial intelligence, thus completing this update of progress in pediatric urology in the early twenty-first century.

We are grateful to all who contributed so generously to this effort and hope the readers will enjoy and profit from this articles as much as we did.

## Author Contributions

All authors listed have made a substantial, direct and intellectual contribution to the work, and approved it for publication.

### Conflict of Interest Statement

The authors declare that the research was conducted in the absence of any commercial or financial relationships that could be construed as a potential conflict of interest.
